# Canva

**DOI:** 10.5195/jmla.2020.940

**Published:** 2020-04-01

**Authors:** Alison Paige Gehred

**Affiliations:** Reference Librarian, Grant Morrow III Library, Nationwide Children’s Hospital, Columbus, OH, Alison.Gehred@nationwidechildrens.org, https://orcid.org/0000-0002-1832-6725

## INTRODUCTION

With the advent of social media, it is no longer enough to merely make print materials to market library services. Libraries often have a social media presence to advertise their programs and raise awareness of what they do. However, these platforms have different parameters of what size and resolution of image they can use, and each is different and constantly being updated. For example, an image that works on Instagram’s grid might not work as well on Instagram Stories.

It is important for the library to maintain a social media presence for several reasons. Social media can highlight social interaction, and staff can get insight into the needs of their users [1]. Premium resources such as Adobe, Corel, or perhaps a Microsoft Office program can be used; however, sometimes stock photos and art do not have a good design aesthetic and can come across as outdated or garish. The production of graphic design materials serves as an extension and reflection of the values of a library institution [2]. It should be taken seriously and help emphasize the library brand. Nancy Stimson, outreach services librarian at University of California San Diego, writes: “A library brand has been defined as ‘all the things that come to mind, all the expectations they have, when they hear the word library, and how you wish people to perceive your library’” [3]. Canva is a product that simplifies the process with templates and other design elements. It is a resource for anyone who wants to create products, from entrepreneurs to educators, to anyone just trying to build a brand. This review discusses the pros and cons of creating designs with Canva and how it can help with the library brand and other library materials.

## CANVA

Canva is a graphic design tool that was created in 2012 by Australian entrepreneur Melanie Perkins. It utilizes a drag-and-drop format that will be familiar to the average user as well as design professionals. It features fonts, graphics, vectors, and templates, and in 2019, the company purchased free stock photo sites Pixabay and Pexels, giving users access to a diverse array of free photos in the program itself. The website also offers photo filters, millions of images, free icons and shapes, and hundreds of fonts. There are thousands of templates to choose from. Some of the more useful ones for librarians might be related to social media, including meme generators, SnapChat geofilters, and Instagram Story templates. Canva also has various marketing templates, including brochures and business cards. Once a design is complete, it can be downloaded in a variety of formats including joint photographic experts group (JPEG), portable network graphics (PNG), and portable document format (PDF). For printing purposes, Canva suggests downloading PDFs in PDF-Print format. Canva offers printing services in 3–5 days. As of December 2019, a t-shirt template has been added. Canva is available in 190 countries and in over 100 languages, and works with all operating systems [4].

New users to Canva Basic can go to canva.com and register for a free account [5]. The app is also available from either the Apple App Store or the Google Play Store. Once designs are uploaded, they can be edited as often as necessary. There are various upgrades, and some content is premium, so users can pay as they go. The basic program does have a very large selection of free photos, illustrations, and vector graphics, but users can upload their own photos as well. Most premium photo prices fall in the $1–$5 range, and as of the writing of this article, all proceeds from photo purchases are going to Australian bush fire relief. Multiple people can work on the same projects in Canva, as the program easily facilitates group work.

### Basic

The basic Canva plan offers 1 gigabyte (GB) of storage for photos and assets, 2 folders to organize designs, access to over 8,000 templates, uploading of personal images, and access to millions of free photos with the option of pay-as-you-go premium content, if needed.

### Canva Pro

Canva Pro offers unlimited folders, team functionalities, 100 GBs of storage for photos and assets, access to over 4 million photos and graphics, custom fonts and a color palette, animated graphics interchange format files (GIFs), priority support, 1-click editing options, saved team templates, search tools, and the ability to resize designs into custom dimensions. This is available free to nonprofits, pending verification of qualifications [6].

### Canva for Enterprise

Canva for Enterprise includes everything in Canva Pro plus unlimited storage, multiple brand kits, the ability to review and comment on team designs, single sign-on support, 99.5% service level agreement (SLA) uptime (basically how often the service is available and operational online), and enterprise support.

## NAVIGATION

Once an account has been created, the home page and navigation system can be utilized. The side panel shows name and profile, the Create a Design button, Home tab, all designs, templates, Design School, Brand Kit, team name, folders, shared items, trash, and upgrade. Brand Kits can be used to manage color schemes and templates for specific branding. Design School offers guides to learn best practices for design. The basic version has a search bar where one can look for templates. Underneath the search bar, there is a listing of personal designs in chronological order. There is also a feature below the created designs that shows recommended design templates. Users can click on a specific design to see a complete listing of templates.

## DESIGN CREATION

According to the creators of Canva, the easiest and best way to learn this program is by experimentation. The program is intuitive and user friendly and offers videos and tutorials in Canva Support. Available tools include templates; elements (shapes, borders, and icons); uploads for personal photos; apps (such as compatibility with DropBox and addition of emojis); a search bar; grids and frames; text and text holders; photos; backgrounds; graphics; and charts.

Once a template is chosen, the drag-and-drop feature can be used to customize a design. The program will resize a selected photo to the correct dimensions, while the text of the template remains in the foreground for immediate use. The program automatically saves any work that is done. When editing the design is complete, it can be downloaded, shared, or printed. For web content, Canva suggests using the PNG file format for image clarity. Users can save multiple versions of designs, edit as needed, make copies, and change the title of the work on the fly.

## USEFUL TOOLS

Canva support is a separate web page with all the resources needed to learn how to navigate the web page, tutorials, forms, and FAQs.

Canva allows users to create more than twenty kinds of professional graphs as well as different ways to embed them, including downloading and adding them to presentations. Canva has several interesting photo-editing tools that go beyond the average filter. It offers features such as a design grid, free icons, stickers, and badges. Canva has an interactive color wheel tool, with which users can create colors, find complementary colors, and learn about different types of color theories. The program also provides blog articles and ideas on how to use templates more creatively. Canva offers several massive online open courses (MOOCs) as part of their Design School. The videos tend to be under ten minutes long and have downloadable content. There are videos on how to integrate design in the classroom as well as the theory and practice of graphic design.

## CONCLUSION

Canva is a comprehensive design tool that is simple to use and wonderful for pros and beginners alike. One of the most useful features is the vast number of easy-to-use templates that can be used for data visualization, marketing, and branding. The drag-and-drop features make the program user-friendly, and the online platform allows users to access their work from multiple devices. The program is intuitive; however, time is needed to learn the program’s unique features. Canva has had its problems, including a massive data breach in 2019. The company has a support FAQ and is working to resolve these problems [7]. The basic program has a huge selection of templates and free photos and is a good value for those libraries that do not want to make a larger investment. Canva is highly recommended for organizations that want to try new and unique ways of designing library materials. Librarians will enjoy using this program and expressing themselves as they create library brands and accompanying materials.

## 

***Alison Paige Gehred, MLIS****, Alison.Gehred@nationwidechildrens.org, https://orcid.org/0000-0002-1832-6725, Reference Librarian, Grant Morrow III Library, Nationwide Children’s Hospital, Columbus, OH*
